# Genome Sequences of 16 Escherichia coli Bacteriophages Isolated from Wastewater, Pond Water, Cow Manure, and Bird Feces

**DOI:** 10.1128/mra.00608-22

**Published:** 2022-09-28

**Authors:** Amira R. Vitt, Stephen J. Ahern, Michela Gambino, Martine C. H. Sørensen, Lone Brøndsted

**Affiliations:** a Department of Veterinary and Animal Sciences, University of Copenhagen, Frederiksberg C, Denmark; Portland State University

## Abstract

Escherichia coli is a highly diverse bacterial species comprising both commensal and pathogenic strains. Here, we report complete genome sequences of 16 E. coli bacteriophages isolated from various environmental samples using the ECOR collection as isolation hosts.

## ANNOUNCEMENT

Escherichia coli is a versatile and genetically diverse species inhabiting the gut of animals and humans as both commensal or pathogenic strains (1). Similarly, E. coli phages are highly diverse currently belonging to 11 different families and 100 different genera (2) and have been isolated from a variety of sources (3–7). Here, we report the complete genomes of 16 phages isolated from diverse environmental sources.

Phages were isolated using all of the 72 strains in the E. coli Reference Collection (ECOR) (8), representing the diversity of E. coli, as isolation hosts. Samples for phage isolation were collected from a variety of sources (i.e., wastewater, pond water, cow manure, and bird feces) in Copenhagen, Denmark, enriched overnight in the presence of ECOR hosts, serially diluted, and 3 drops of 10 μl were spotted on the same host (Table 1). Single plaques were isolated and plaque purified for at least three rounds as described elsewhere (9). Selected phage genomic DNA was isolated from 1 mL crude phage lysate by using the standard phenol-chlorophorm extraction followed by ethanol precipitation protocol (10). Genomic DNA libraries were prepared using Nextera XT kit and sequenced using Illumina MiSeq platform (250 bp paired end). The raw reads were trimmed and used for *de novo* genome assembly with CLC Genomics Workbench 9.5.3 (Qiagen, Århus). The assembled genomes were annotated using Rapid Annotations using Subsystems Technology (RAST) (11). All tools were run with default parameters.

**TABLE 1:** Properties of 16 Escherichia coli bacteriophage genomes

Name	Isolation host	Isolation source	Family	Genus	Total read count	Avg coverage	Genome length (bp)	ORFs^*a*^	tRNA	G+C (%)	Accession	Closest relative	Coverage (%)	Identity (%)	Accession
EC120	ECOR4	Wastewater	*Autographiviridae*	*Vectrevirus*	674959	3327	44544	55	ND^*b*^	44.9	ON185580.1	Escherichia phage vB_EcoS_FFH_1	90	96.09	KJ190157.1
EC100	ECOR4	Pond water	*Demerecviridae*	*Tequintavirus*	222491	433	108723	155	19	39	OK665835.1	Escherichia phage vB_EcoS_FFH_1	90	96.09	KJ190157.1
EC104	ECOR19	Bird feces	*Demerecviridae*	*Tequintavirus*	163981	382	108711	155	19	39	ON185581.1	Escherichia phage vB_EcoS_FFH_1	90	96.09	KJ190157.1
EC105	ECOR20	Bird feces	*Demerecviridae*	*Tequintavirus*	150574	319	108732	155	19	39	ON185582.1	Escherichia phage vB_EcoS_FFH_1	90	96.08	KJ190157.1
EC122	ECOR20	Bird feces	*Demerecviridae*	*Tequintavirus*	360008	636	108723	156	19	39	ON185583.1	Escherichia phage SP15	90	92.08	NC_048627.1
EC142	ECOR4	Bird feces	*Demerecviridae*	*Tequintavirus*	123228	204	108723	155	19	39	ON185584.1	Escherichia phage vB_EcoS_FFH_1	90	96.09	KJ190157.1
EC148	ECOR4	Bird feces	*Demerecviridae*	*Tequintavirus*	71380	187	107754	157	19	39	ON185585.1	Escherichia phage phiLLS	92	92.49	NC_047822.1
EC125	ECOR13	Wastewater	*Drexlerviridae*	*Warwickvirus*	534498	2617	50477	83	ND	44.6	ON185586.1	Escherichia phage tuinn	97	98.72	MN850606.1
EC167	ECOR20	Wastewater	*Drexlerviridae*	*Warwickvirus*	487226	1733	50988	82	ND	44.2	ON185587.1	Escherichia phage vB_EcoS-95	88	88.45	NC_048132.1
EC195	ECOR34	Pond water	*Drexlerviridae*	*Warwickvirus*	171094	485	50477	83	ND	44.6	ON185588.1	Escherichia phage tuinn	97	98.76	MN850606.1
EC147	ECOR4	Wastewater	*Drexlerviridae*	*Hanrivervirus*	587499	3008	46060	77	ND	43.9	ON185589.1	Escherichia phage egaa	95	95.98	NC_049852.1
EC101	ECOR4	Pond water	*Straboviridae*	*Tequatrovirus*	201824	270	167718	264	10	35.4	OL310488.1	Escherichia phage vB_EcoM-G3F9	98	99.12	MZ234037.1
EC128	ECOR20	Pond water	*Straboviridae*	*Tequatrovirus*	61340	83	171814	270	8	35.3	ON210139.1	Escherichia phage vB_EcoM_112	94	97.43	KJ668714.2
EC106	ECOR4	Wastewater	None	*Felixounavirus*	27683	73	87829	128	20	39.1	ON210138.1	Escherichia phage tootiki	91	96.94	MN850647.1
EC150	ECOR36	Cow manure	None	*Wifcevirus*	601535	2148	68034	94	ND	46.1	ON210137.1	Escherichia phage vB_EcoM-Ro157lw	96	97.53	MH051335.1
EC115	ECOR20	Cow manure	None	*Dhillonvirus*	299207	1542	44909	63	ND	54.6	ON210136.1	Escherichia phage vB_EcoS_Over9000	98	94.33	OK499985.1

a ORFs, open reading frames.

b ND, no tRNAs were identified by RAST annotation.

Phage genera were preliminary assigned by overall genome BLAST similarities to the closest related phage genome available at the NCBI and stated in Table 1. The most diverse phages were isolated from pond water and wastewater, representing four different families, with phages from more genera represented in the wastewater.

The genomes ranged in size from 44544 bp (EC120) to 171814 bp (EC128, EC150) encoding 55 to 271 predicted open reading frames (Table 1). The G+C content ranged from 35.3% (EC128) to 54.6% (EC115). Also, the number of predicted tRNAs varied between the phages (none to 20 tRNAs). The largest number of the predicted tRNAs was observed in phage EC106.

All isolated phages showed similarity to known E. coli phages, determined by whole-genome BLAST similarity search at the NCBI database and stated in Table 1. Phage EC167 was the least similar to other coliphages with 88% query coverage and 88.5% sequence identity to phage vB_EcoS-95 (NC_048132). Phage EC148 showed the highest sequence homology to phage T5_ev212 (99.97% sequence identity, 99% query coverage). For the remaining phages in the collection, closely related phages had between 90 and 96% sequence identity over 90 to 93% query coverage. In summary, while many of the E. coli phages in our collection were closely related to the known E. coli phages, a few phages showed limited sequence similarity, thus expanding the knowledge on diversity of E. coli phages.

### Data availability.

The raw sequence reads are available at NCBI SRA database with accession number SRP394492 and GenBank accession numbers for the 16 phages are listed in Table 1.
